# microRNA-451a regulates colorectal cancer proliferation in response to radiation

**DOI:** 10.1186/s12885-018-4370-1

**Published:** 2018-05-03

**Authors:** Rebecca Ruhl, Shushan Rana, Katherine Kelley, Cristina Espinosa-Diez, Clayton Hudson, Christian Lanciault, Charles R. Thomas, V. Liana Tsikitis, Sudarshan Anand

**Affiliations:** 1 Department of Cell, Developmental & Cancer Biology, Oregon Health & Science University, 3181 SW Sam Jackson Park Road, Portland, OR 97239 USA; 2 Department of Radiation Medicine, Oregon Health & Science University, 3181 SW Sam Jackson Park Road, Portland, OR 97239 USA; 3 Department of Surgery, Oregon Health & Science University, 3181 SW Sam Jackson Park Road, Portland, OR 97239 USA; 4 Department of Pathology, Oregon Health & Science University, 3181 SW Sam Jackson Park Road, Portland, OR 97239 USA

**Keywords:** microRNAs, Colorectal cancer, Radiation therapy, CAB39, EMSY

## Abstract

**Background:**

Colorectal cancer (CRC) is a leading cause of cancer-related death. The biologic response of CRC to standard of care adjuvant therapies such as chemotherapy and radiation are poorly understood. MicroRNAs (miRs) have been shown to affect CRC progression and metastasis. Therefore, we hypothesized that specific miRs modulate CRC response to chemoradiation.

**Methods:**

In this study, we used miR expression profiling and discovered a set of microRNAs upregulated rapidly in response to either a single 2 Gy dose fraction or a 10 Gy dose of γ-radiation in mouse colorectal carcinoma models. We used gain and loss-of-function studies in 2D and 3Dcell proliferation assays and colony formation assays to understand the role of the top miR candidate from our profiling. We used Student’s T-tests for simple comparisons and two-factor ANOVA for evaluating significance.

**Results:**

The most upregulated candidate at early time points in our signature, miR-451a inhibited tumor cell proliferation and attenuated surviving fraction in longer-term cultures. Conversely, inhibition of miR-451a increased proliferation, tumorsphere formation, and surviving fraction of tumor cells. Using a bioinformatics approach, we identified four genes, CAB39, EMSY, MEX3C, and EREG, as targets of miR-451a. Transfection of miR-451a decreased both mRNA and protein levels of these targets. Importantly, we found miR-451a expression was high and CAB39, EMSY levels were low in a small subset of rectal cancer patients who had a partial response to chemoradiation when compared to patients that had no response. Finally, analysis of a TCGA colorectal cancer dataset revealed that CAB39 and EMSY are upregulated at the protein level in a significant number of CRC patients. Higher levels of CAB39 and EMSY correlated with poorer overall survival.

**Conclusions:**

Taken together, our data indicates miR-451a is induced by radiation and may influence colorectal carcinoma proliferation via CAB39 and EMSY pathways.

**Electronic supplementary material:**

The online version of this article (10.1186/s12885-018-4370-1) contains supplementary material, which is available to authorized users.

## Background

In 2016, an estimated 134,000 patients will be diagnosed with colorectal cancer in the United States. Among the rectal cancer subset, patients with locally advanced disease, stages T3-T4, node positive, receive neoadjuvant chemoradiation therapy (CRT) and subsequent surgery [1, 2]. The standard of care is still surgical resection with total mesorectal excision that may result in significant quality of life issues [3]. Despite neoadjuvant chemoradiation, patients may still have residual disease. Response to CRT is an independent predictor of overall survival in colorectal cancer [4] highlighting the need for improved CRT response rates. It is known that several tumor intrinsic factors govern responses to CRT, including specific gene expression programs with distinct significance ascribed to microRNAs (miRs) [5, 6]. miR-processing machinery is frequently mutated in colorectal cancers (TCGA, 2016 provisional), and miRs have been implicated in several pathological processes associated with colorectal cancer progression, including cancer stemness and epithelial-to-mesenchymal transition (EMT) [7, 8]. Emerging evidence suggests that microRNAs (miRs) modulate gene expression programs in response to radiation and confer variable sensitivity and efficacy to modern high dose ionizing radiation therapy [9, 10]. In this context, we have identified miR-451a as an anti-proliferative microRNA in colorectal adenocarcinoma whose presence correlates with increased radiation efficacy. Through gain and loss-of-function studies, we show that miR-451a is a negative regulator of proliferation in CRC and likely mediates its effects by targeting CAB39 and EMSY. We believe our work highlights the potential for using miRs and their target genes in predicting radiation responsiveness of CRC while also illustrating potential avenues for restoring radiation sensitivity in poorly responding tumors.

## Methods

### RNA extraction, RT-PCR, miR profiling

RNA was extracted using the miRVana microRNA isolation kit (Ambion/Life Technologies). Affymetrix microRNA array v4.0 profiles were generated by the OHSU Genome Profiling Core facility. Individual RT-PCRs were performed using predesigned TaqMan Assays for mature miRs, primary miRs or mRNAs (Applied Biosystems) on a Vii-7 real time PCR platform (Applied Biosystems) according to manufacturer’s instructions. Data was normalized to internal control small RNA RNU48 or U6 small RNAs. mRNAs were normalized to either β-actin or GAPDH.

Nanostring microRNA profiling was performed per manufacturer’s instructions and data was analyzed using the N-solver software. Raw data was normalized to housekeeping genes.

### Cell culture and reagents

HCT-116 cells (ATCC) were cultured in McCoy’s supplemented with 10% FBS and antibiotics. CT26 cells (ATCC) were cultured in RPMI-1640 medium supplemented with 10% FBS and antibiotics. SW480 and SW620 cells (ATCC) were cultured in Leibovitz’s L-15 Medium with 10% FBS, L-glutamine, antibiotics under 0% CO_2_ conditions. Human Umbilical Vein Endothelial Cells (HUVECs, Lonza) were cultured in EGM2 media with 10% FBS and all growth factors provided with the EGM2 bullet kit. Normal human lung fibroblasts (ATCC) were cultured in DMEM with 10% FBS. Cells were tested and found negative for mycoplasma contamination before use in the assays described.

### Transfections

Cells were reverse transfected with miR-451a-5p mimics, inhibitors, selected siRNAs against CAB39 and EMSY and their respective controls purchased from Life Technologies using Lipofectamine RNAiMAX (Invitrogen) according to manufacturer’s instructions.

### Colony formation assay

Cells were transfected with miR-451a-5p mimic or control mimic for 16 h. Then cells were plated (100 or 200 cells for 0Gy, 200 or 400 cells/well for 2GY and 400 or 800 cells per well for 5Gy) in triplicate in a 6 well plate. Cells were irradiated 24 h after plating, 0Gy, 2Gy or 5Gy. Two weeks after plating, cells were fixed and stained with crystal violet and colonies were counted. For each condition, cells were plated in two different densities in triplicate and mean colony number was used for calculations. Plating efficiency was calculated as 100 * (No. of colonies counted/ No. of cells plated). Surviving fraction was calculated as plating efficiency of treated/plating efficiency of untreated sample.

### In vivo assays

All animal work was approved by the OHSU Institutional Animal Use and Care Committee. Animal experiments were performed in accordance with the OHSU IACUC guidelines and regulations. Immune compromised 8–10 week old female nu/nu mice purchased from Jackson Labs were injected subcutaneously with 1 million mycoplasma-negative tumor cells in Matrigel (BD) per flank. 8–10 week old female Balb/C mice were injected subcutaneously with 1X10^4^ CT26 cells. Tumor growth was measured with calipers, with volume computed as ½ * Length * Width^2^. Mice were randomized into groups once the average tumor volume reached 80mm^3^, approximately 6 days after injection. Mouse CD8 T-cells were purified from pooled spleen and lymph node single cell suspensions of Balb/C or C57BL/6 mice (*n* = 6 mice) using negative selection based magnetic beads (Biolegend) per manufacturer’s recommendations.

### Irradiation

Cells or mice were irradiated on a Shepherd^137^cesium irradiator at a rate of B166 1.34 cGy min. In tumor-targeted radiation experiments, mice were restrained in a lead shield (Brain Tree Scientific) to minimize exposure to the non-tumor areas.

### Cell titer Glo/ caspase Glo

HCT-116 cells were transfected in a 6 well plate with miR-451a-5p mimic or inhibitor, and the corresponding controls from Life Technologies as previously described. Cells were transferred to a 96 well plate 16 h post-transfection (1000 cells/well). At 24 h post-transfection the HCT-116 cells were irradiated with 0, 2, or 5 Gy. Cell Titer-Glo and Caspase 3/7 Glo were analyzed at 48 h and 96 h post-irradiation, according to manufacturer’s instructions.

### miR-Trap

293 T cells were co-transfected with a plasmid coding for a flag-tagged dominant negative GW418 mutant (Clontech kit #632016) along with a control mimic or miR-451a-5p mimic according to manufacturer instructions. Twenty-four hours later the RNA protein complexes were crosslinked and the RISC complex was immunoprecipitated using an anti-FLAG antibody and RNA was isolated for quantitative real-time PCR of target genes. The fold enrichment was calculated using pre and post immunoprecipitation (IP) controls as well as normalization to the control mimic pull-downs.

### Western blotting

Cell lysates were prepared in RIPA buffer (Pierce 89,900) and quantified using a BCA assay (Pierce, #23227) kit. Equivalent amounts of protein were loaded on a 4–15% gradient SDS-polyacrylamide gel (Mini-PROTEAN TGX Precast Gels, BioRad) and transferred onto Nitrocellulose membranes using TransBlot Turbo (BioRad). Membranes were blocked in 5% milk and incubated with antibodies as indicated- CAB39 (Genetex #110628, 1:500 o/n), EMSY (Abcam, #32329 1:300 o/n), Anti-β-actin antibody (Sigma, A5316, 1:10,000 1 h RT). Membranes were washed in TBST and incubated with appropriate secondary antibodies from Licor Biosciences (1:15000). Blots were scanned on the Licor Odyssey scanner according to manufacturer’s instructions.

### Patient tissue collection

Patients were identified with a rectal cancer diagnosis from the years 2000 to 2016 in the Oregon Colorectal Cancer Registry (OCCR) [11]. The registry is maintained by Salem Hospital and the Oregon Health & Sciences University and catalogs patients treated for colorectal cancer at both institutions. OHSU Institutional Research Board (IRB) approval was granted for the study, and a written informed consent was obtained from all participating patients. Pre-treatment formalin-fixed paraffin-embedded (FFPE) specimens, post-treatment surgical FFPE specimens, and plasma are stored on-site. Patients were categorized as either non-, partial, or complete pathological responders, based on a pathological tumor regression score. Non-responders had greater than 50% tumor, partial responders had less than 50%, on pathologic review [12].

Pre-treatment and post-treatment specimens were prepared on 5 μm thick FFPE slides, which were assessed independently by two separate pathologists. Pre-treatment specimens were used for miR qRT-PCR experiments. Slides containing greater than 20% necrosis were not utilized for study.

## Results

### Early and late radiation responsive miRs in CRC

**Fig. 1:**
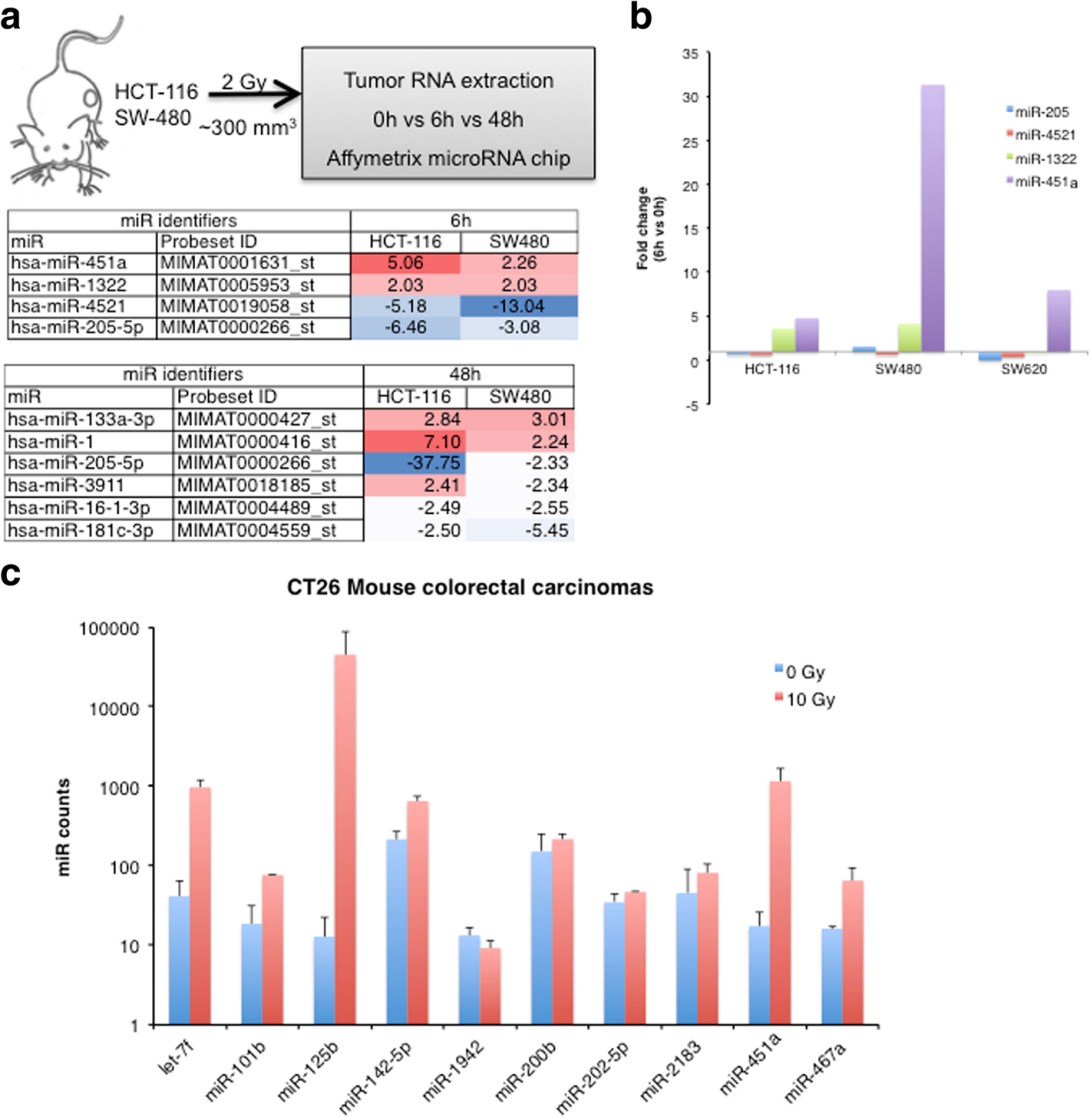
miR-451a is robustly induced by radiation in human and mouse colorectal carcinoma. **a** Design of the screen for miRs induced by radiation. Heatmap depicts miRs with more than 2 fold change from the affymetrix microRNA array v4.0 across both tumor types. **b** Levels of the two most upregulated and downregulated miRs 6 h post 2 Gy radiation were validated by qRT-PCR using specific Taqman probes for each microRNA. Mean fold change after normalization to a housekeeping RNA, RNU48, is depicted. **c** Most abundant (> 10 counts) miRs in CT26 mouse colorectal carcinomas at 6 h post 10 Gy treatment. Bars represent mean + SD of 3 mice in 0 Gy group and 2 mice in 10 Gy group. RNA was extracted from tumors and miR profiles were analyzed using the Nanostring miR panel

To identify miRs that are regulated by radiation in CRC, we implanted either HCT-116 or SW480 xenografts into nude mice. We chose these two cell lines because they have been well-characterized and grow well in vitro and in xenograft studies [13, 14]. In addition, they provide a contrast of sorts with HCT-116 being microsatellite unstable (MSI), PIK3CA H1047R mutated and p53 WT whereas SW480 is microsatellite stable (MSS), PIK3CA WT but p53 R237H mutated. After the tumors reached a 300mm^3^ volume, we treated the mice with a single 2 Gy focal radiation. Tumors were harvested at either 6 h or 48 h post-RT and RNA was extracted to generate the initial in vivo miR profile using Affymetrix microRNA arrays (Complete datasets in Additional file 1). Using a 2-fold regulation in both cell lines as a cut-off, we identified two miRs that were upregulated and two miRs that were downregulated at 6 h (Fig. 1a). We focused on the miRs induced at the 6 h time point since direct transcriptional effects are apparent at this time point and there could be significant secondary transcriptional and cell cycle dependent effects at 48 h. The top candidate in this profile, miR-451a was validated using qRT-PCR across three different human CRC cell lines (Fig. 1b) grown as subcutaneous xenografts. Importantly, the induction of miR-451a was equally robust in a CT26 mouse colorectal carcinoma model in response to a 10 Gy dose of focal radiation (Fig. 1c). Surviving fractions assays (Additional file 2: Figure S1) indicate that both CT26 and HCT-116 cells have decrease in survival at higher doses of radiation. Indeed, miR-451a induction correlated with the dose of radiation in both HCT-116 and CT26 cell lines with maximal expression at higher doses (Additional file 2: Figure S2). We asked if induction of miR-451a was a response unique to malignant cells or whether normal cells responded similarly to radiation. We evaluated the induction of miR-451a in three different non-transformed cells -human umbilical vein endothelial cells (HUVECs), normal human lung fibroblasts (NHLFs) and mouse naïve primary CD8 T-cells. Of these, we observed that the miR-451a expression was at the detection threshold in the fibroblasts and mouse CD8 T-cells and did not increase appreciably with radiation. However, HUVECs induced miR-451a in a dose responsive manner (Additional file 2: Figure S3).

### miR-451a inhibits cell proliferation in colorectal cancer

**Fig. 2:**
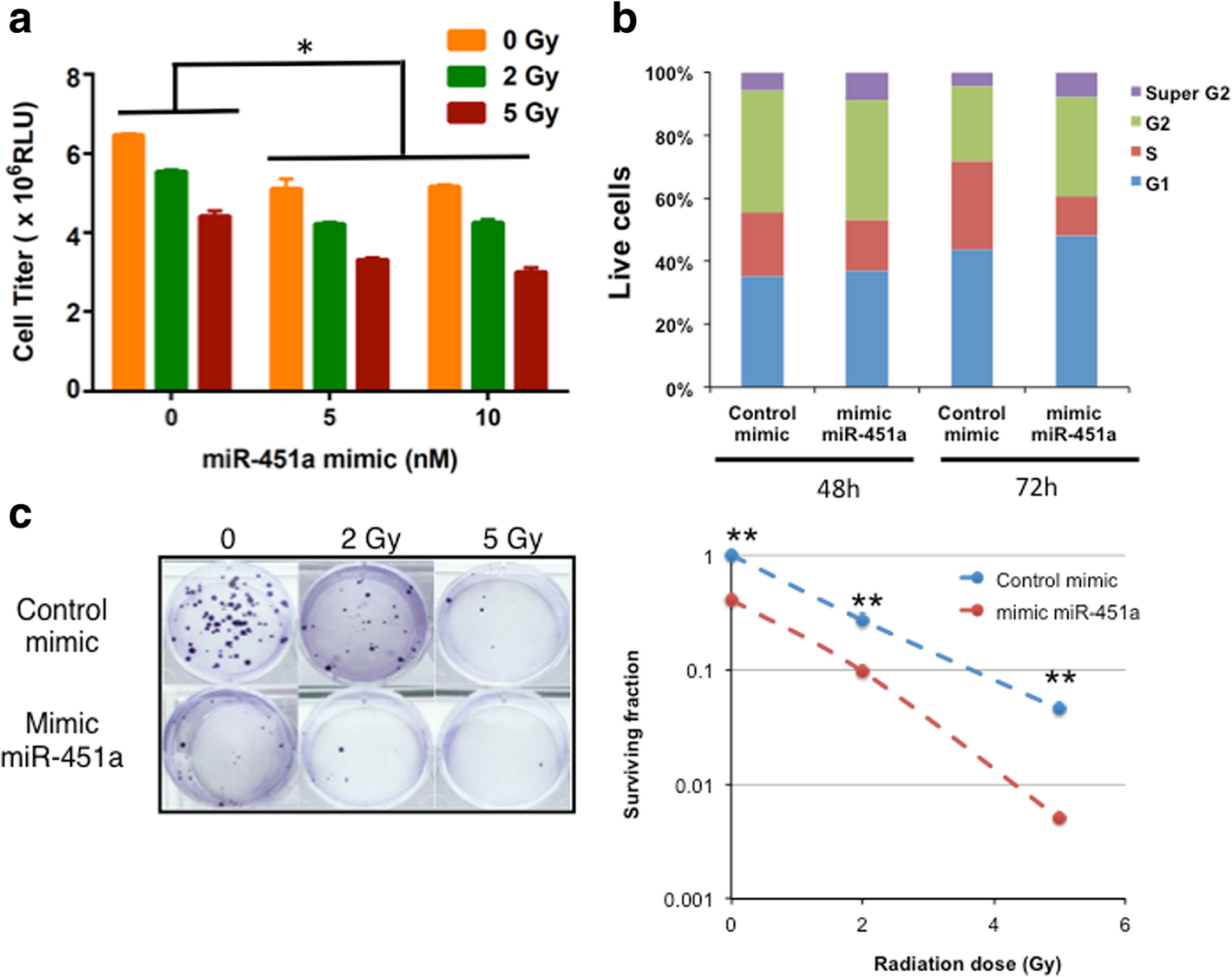
Ectopic expression of miR-451a inhibits proliferation and clonogenic survival of HCT-116 cells. A) HCT-116 were transfected with a miR-451a mimic or a control mimic at the indicated doses. Proliferation was analyzed 48 h after radiation with the indicated doses. Bars depict mean ± s.e.m. of triplicate wells. * indicates *P* < 0.05 on ANOVA for interaction between radiation and miR treatments. **b** HCT-116 cells were transfected as in **a** and cell cycle analysis was performed via flow cytometry of propidium iodide stained cells at the indicated time points. **c** HCT-116 cells were transfected and irradiated as described in A and plated 24 h later. 12–14 days after plating, cells were fixed and stained with crystal violet and colonies were counted. Surviving fraction was calculated based on the colony numbers normalized to the plating efficiency. Mean of triplicate wells is plotted. * indicates *P* < 0.05 and ** indicates *P* < 0.01on a two-tailed Student’s T-test

To understand the function of miR-451a, we first performed gain of function studies in vitro with HCT-116 cells. Ectopic expression of low doses of miR-451a decreased proliferation in 2D culture modestly (Fig. 2a). Combination of radiation with low dose miR-451a resulted in stronger effects on proliferation (Fig. 2a). The combination index (CI) calculations using the Chou-Talalay method [15] indicated values ranging from 0.42 (synergistic interaction 5 Gy + miR-451a) to 0.8 (additive effect 2 Gy + miR-451a). Similarly, transfection of the mimic resulted in a slight decrease in S-phase cells at 48 h and a more pronounced decrease at 72 h post transfection as measured by cell cycle analysis using propidium iodide (PI) staining (Fig. 2b). This data suggests that miR-451a transfected cells are still vulnerable to radiation within 24-48 h after transfection and possibly have other complementary mechanisms that result in proliferation arrest at the later time points. We then asked if these effects were viable over long-term culture in surviving fraction studies. We first validated that our transfected cells retained expression of the miR over 9 days in colony formation assays (Additional file 2: Figure S4). We note that while miR-451a alone had a significant effect on the phenotypes, there was also synergistic effect with radiation at the 5 Gy dose (Fig. 2c, CI < 0.3 for miR-451a and 5 Gy). This inhibition of proliferation and clonogenic survival was also confirmed in CT26 cells (Additional file 2: Figure S5). In both the short-term and long-term cultures, interaction of miR with radiation was deemed to be statistically significant by two-factor ANOVA (*P* < 0.05).

**Fig. 3:**
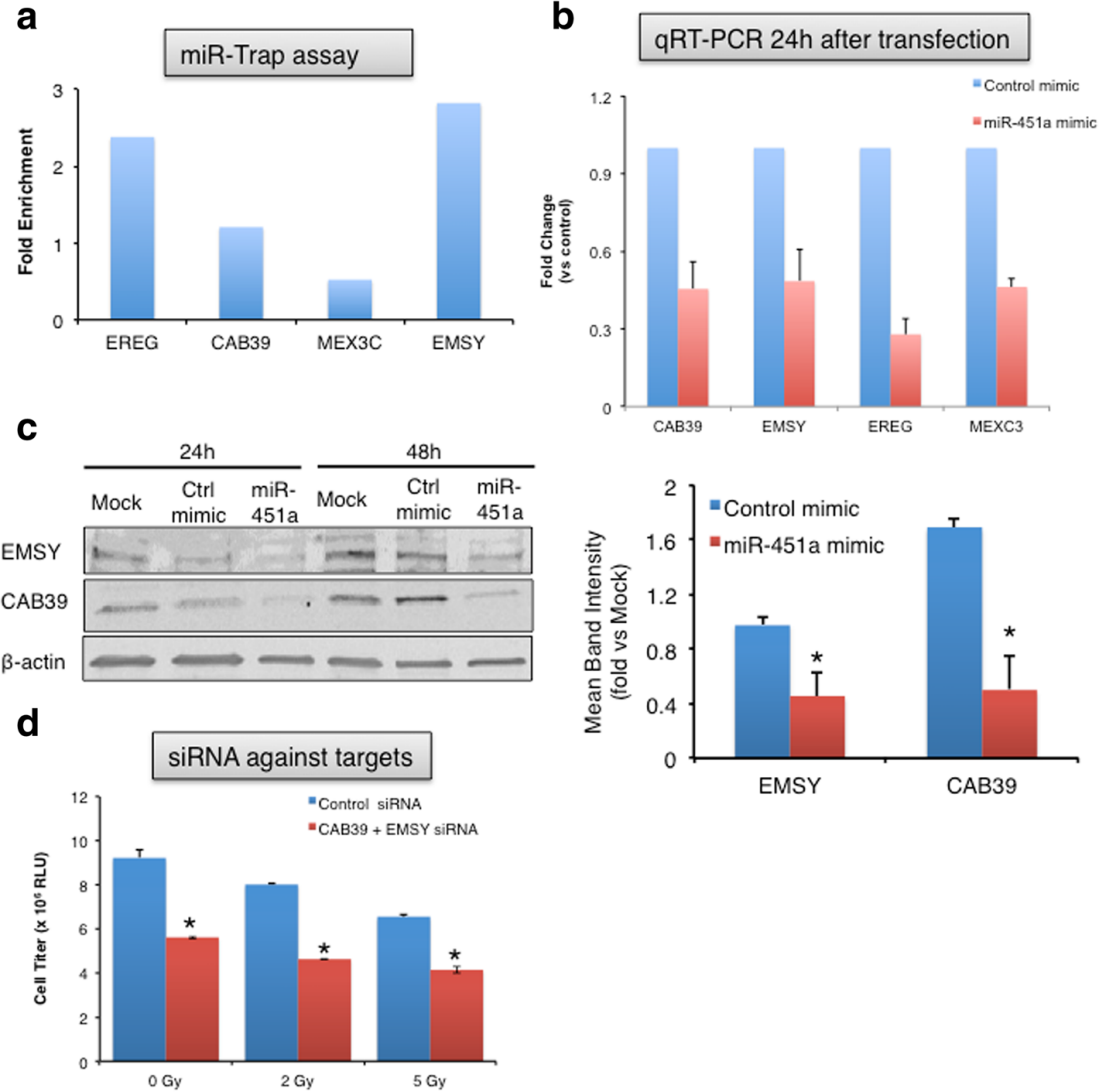
miR-451a inhibition increases tumor cell proliferation and clonogenic survival. HCT-116 were transfected with a miR-451a inhibitor (anti-miR-451a) or a control anti-miR. Proliferation was analyzed 48 h after radiation in **a** 2D and **b** 3D cultures with the indicated doses. Bars depict mean ± s.e.m. of triplicate wells. ** indicates *P* < 0.01 on a two-tailed Student’s T-test. **c** 12–14 days after plating, cells were fixed and stained with crystal violet and colonies were counted. Surviving fraction was calculated based on the colony numbers normalized to the plating efficiency. Mean of triplicate wells is plotted. * indicates *P* < 0.05 and ** indicates *P* < 0.01on a two-tailed Student’s T-test

Conversely, inhibition of miR-451a enhanced proliferation in 2D (Fig. 3a) and tumorsphere assays (Fig. 3b) almost negating the effects of a 2 Gy dose of radiation. The preservation of survival was most robust in the 2 Gy population with a log fold increase in clonogenic survival, but was not evident at higher dose radiation (Fig. 3c). Inhibition of miR-451a had modest effects on proliferation in HUVECs (Additional file 2: Figure S6). In contrast to radiation, miR-451a transfection did not appreciably increase the responses to 5-FU, a commonly used radiosensitizer in rectal cancer (Additional file 2: Figure S7). Taken together, these studies indicate that miR-451a regulates proliferation of colorectal cancer cells.

### miR-451a targets genes involved in metabolism and DNA repair pathways

**Fig. 4:**
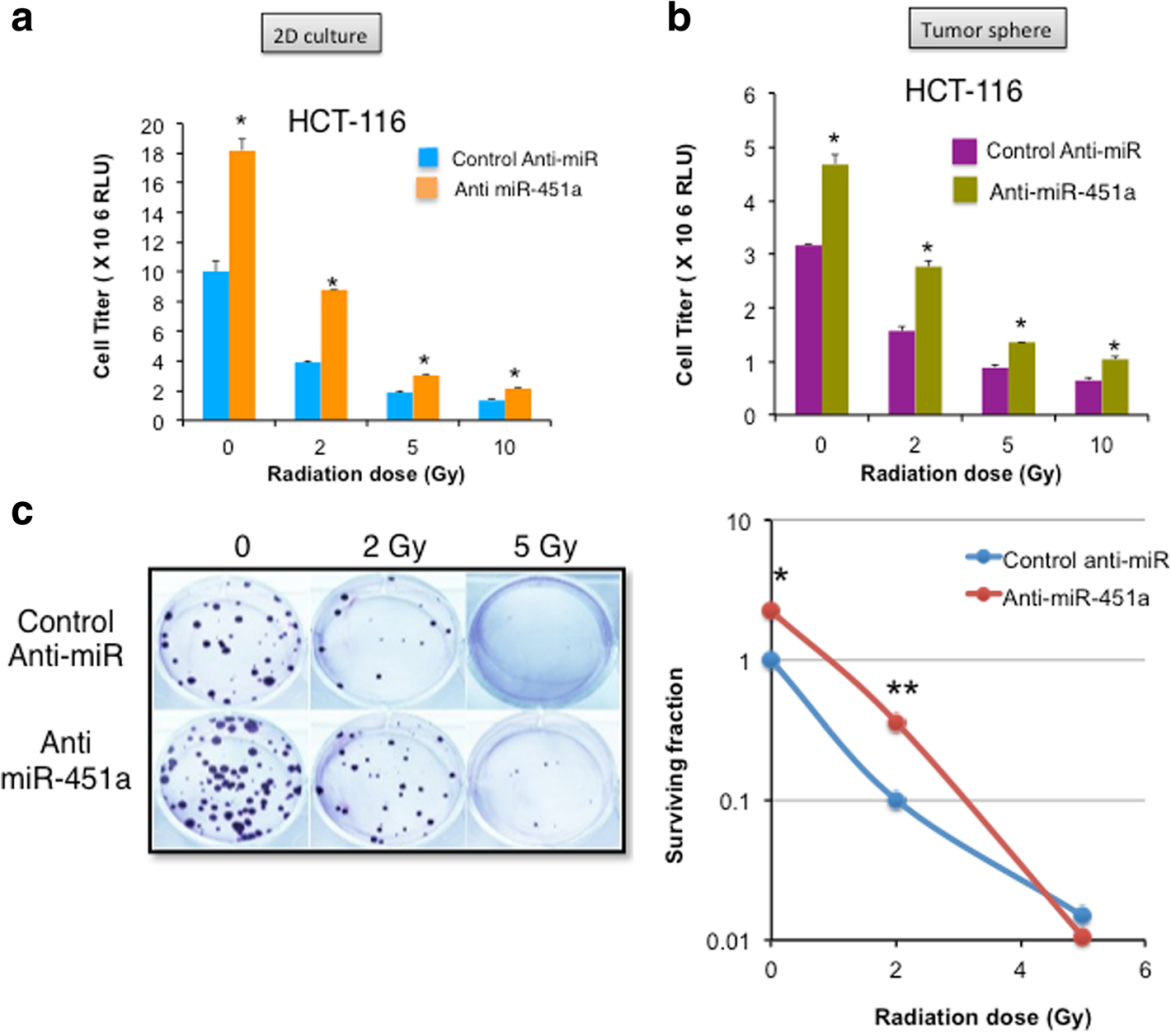
miR-451a targets genes involved in cell cycle and cellular stress responses. **a** miR-TRAP assay depicting enrichment of target mRNAs immunoprecipitated from HCT-116 cells co-transfected with a mutant RISC complex plasmid and a miR-451a mimic or a control miR mimic. Fold enrichment over pre-IP mRNAs is depicted. One of two independent experiments. **b** qRT-PCR of the miR-451a targets in HCT-116 cells at 24 h after transfection. **c** Western blot for EMSY and CAB39 in HCT-116 cells at 24 h and 48 h after transfection of miR-451a compared to control mimic or mock transfection. Right panels show quantitation of band intensity at 48 h from two independent experiments. **d** siRNA mediated silencing of EMSY and CAB39 phenocopies the miR-451a effects in HCT-116 proliferation. * *P* < 0.05, Student’s T-test

To elucidate the relevant miR-451a targets in relation to radiation and colorectal cancer, we used the miRwalk algorithm to combine data from multiple target prediction databases and identified 13 genes as putative targets of miR-451a (NSMAF, OSR1, PMM2, POU3F2, SMARCA2, ARHGEF3, CAB39, ZNF644, CPD, EREG, GRSF1, MEX3C, EMSY). We further narrowed this list by filtering genes with a known role in human colorectal cancer and/or cellular response to ionizing radiation that resulted in a group of four genes -CAB39, EMSY, EREG, and MEX3C. We identified binding sites for miR-451a on all four target mRNAs (Additional file 2: Figure S8). We used a miR-Trap assay to evaluate the target mRNAs bound by miR-451a. The miR-Trap assay involves transfection of a FLAG-tagged dominant negative GW182 (a component of the RNA induced silencing complex, RISC) along with miR-451a or a control miR into 293 T cells. This dominant negative protein is incorporated into RISC and prevents miR bound mRNAs from getting degraded. Therefore 24 h after transfection, cross-linking and an anti-FLAG pull-down enriches for all target mRNAs bound to a miR compared to a control miR. We found that these four target mRNAs were enriched at the RISC with EMSY and EREG more abundant than CAB39 and MEX3C (Fig. 4a). Transfection of HCT-116 cells resulted in significant downregulation of all four target genes at the mRNA level (Fig. 4b). A decrease in gene expression levels after miR transfection can be either directly due to miR-mediated degradation or indirectly due to transcriptional or other posttranscriptional mechanisms triggered by the miR. There is also a possibility of some targets being more resistant to recruitment to the dnGW182. Therefore, we chose to focus on CAB39 and EMSY which were both enriched in the miR-Trap assay and downregulated in qRT-PCR. We confirmed that the mRNA downregulation resulted in a decrease in protein expression for CAB39 and EMSY (Fig. 4c) at 48 h as quantified in the bar graphs (Fig. 4c, right panel). Moreover, siRNA mediated silencing of two of the four targets EMSY and CAB39 in HCT-116 cells recapitulated the miR-451a induced inhibition of proliferation (Fig. 4d). These experiments suggest that miR-451 may decrease EMSY and CAB39, which could mediate the decrease in cell proliferation.

### miR-451a and target expression in colorectal cancer patients

To assess the relevance of miR-451a in human colorectal cancer, we measured the miR levels in pretreatment biopsies from a small group of rectal cancer patients. Patients with partial response (as defined by pathologic regression score) had higher levels of miR-451a as well as lower levels of both CAB39 and EMSY (Additional file 2: Figure S9a). Moreover, analysis of the TCGA database (Provisional colorectal carcinoma, *n* = 633 patients) showed that CAB39 and EMSY protein levels were found to be upregulated in 14 and 6% of cases, respectively (Additional file 2: Figure S9b). Interestingly, upregulated expression of these genes correlated with poorer overall survival (Additional file 2: Figure S9c) at moderate statistical significance.

## Discussion

Several recent studies have shown the utility of miRs in the diagnosis and classification of CRC [7, 8, 16–19]. There are ongoing prospective clinical trials evaluating miR based classifiers such as a 24-miR signature in lung cancer diagnosis [20] (Gensignia) and a miR signature in prostate cancer screening [21] (Exiqon). These trials highlight the feasibility and translational potential of miR-based classifiers. We identified a group of miRs that are responsive to radiation in mouse tumor models and focused on characterizing miR-451a as one of the robust early response miRs in CRC. Our data suggests that miR-451a behaves as an anti-proliferative miR in CRC cell lines in vitro. We also identify putative targets of miR-451a and show that expression of both the miR and two of the targets correlate with radiation responses in CRC. Taken together, our observations suggest that miR-451a modulation of CAB39 and EMSY target genes could alter radiation sensitivity of human CRC.

miR-451a has been found to inhibit cell proliferation and drug responses in other malignancies. For example, miR-451a was found to affect proliferation and sensitivity to tamoxifen in breast cancer via targeting of the macrophage migration inhibitory factor (MIF) [22]. Similarly, miR-451a was shown to be tumor suppressive in gastric cancer by affecting the PI3K/mTOR pathway [23]. In other tumor types, it has been shown that miR-451a expression is downregulated in the tumor cells in a manner consistent with a tumor suppressor function [24, 25]. Interestingly, miR-451a appears to increase radiation responses in nasopharyngeal carcinoma cells [26] and lung adenocarcinoma cells [27]. Our observation in CRC cell lines suggests a function similar to the tumor suppressive role that has been documented in other cancers by these studies.

Using a bioinformatics approach, we narrowed down the targets of miR-451a to a group of 14 genes, which was further filtered to 4 genes –CAB39, EMSY, EREG and MEX3C based on either a known role in colorectal cancer or radiation responsiveness in other cancer types. Calcium binding protein (CAB) 39 has been previously shown to be a target of miR-451a in human glioma [28] and in colorectal cancer [29]. This protein is thought to affect STK11 activity and localization thereby influencing the PI3K/AKT signaling pathway. EMSY is a transcriptional repressor that associates with BRCA2 and is often amplified in breast and ovarian cancers [30]. Functionally, EMSY colocalizes and forms foci with histone γH2AX in response to irradiation. Importantly, breast cancer patients with EMSY amplifications have poorer overall survival. Taken together, these functions suggest that modulation of EMSY by miR-451a may have significant impact on radiation responses and tumor cell survival. Indeed, consistent with the breast cancer dataset, our analysis of the TCGA colorectal cancer dataset (Additional file 2: Figure S9b-c) indicates that EMSY as well as CAB39 are increased at the protein level in a subset of patients and associated with worse outcome. Epiregulin (EREG) is a known ligand of the EGF family and regulates several key processes including cellular proliferation, inflammation, angiogenesis and wound healing [31]. While it has been proposed as a biomarker for monitoring responses to cetuximab in colorectal cancer [32], it is unclear whether there is a function for EREG specifically in the context of radiation responses in these tumors. MEX3C has been identified as a ubiquitin ligase as well as an RNA binding protein that modulates the levels of HLA-A allotypes [33]. Interestingly, it is part of a group of genes that suppress chromosomal instability in colorectal cancer [34] thereby likely contributing to tumor drug resistance. Our data suggests that miR-451a mRNA binds to all four of these mRNAs (Fig. 4a) and downregulates their expression at the RNA and protein levels. Given the low expression levels of the EREG and MEX3C in patient samples, and the discordance between our miR-Trap assay and qRT-PCR, we chose to focus on CAB39 and EMSY. CAB39 and EMSY upregulation worsened CRC survival in the TCGA. We also noticed a trend towards decreased miR expression in patients with more advanced disease compared to adjacent normal tissue (data not shown). We must emphasize that our small patient numbers preclude drawing stronger conclusions regarding the miR-451a and target levels in human disease, but rather lead us to hypothesize that tumors with increased miR-451a levels may respond better to CRT, improving survival.

## Conclusions

Our work demonstrates that miR-451a is a radiation-induced miR in CRC and identifies novel targets of miR-451a that may contribute to radiation responses. Further work is required to validate these observations in larger numbers of patients as well as elucidate the mechanistic basis of miR-451a mediated decrease in proliferation via these target pathways. We envision that our data herein will enable the development and validation of miR and/or target biomarkers that predict radiation responsiveness as well as provide strategies for enhancing the effectiveness of chemoradiation in colorectal cancers.

## Additional files

Additional file 1: **Figure S1** Affymetrix .cel files were uploaded to Partek Genomics Suite 6.6 and normalized using the default parameters of the RMA subroutine (background correction, quantile normalization, median polish summarization) as a single set. All probesets on each array were included in normalization. Following normalization, the log2 transformed signal data set was filtered to exclude all non-human probesets and control probesets. These miRNAs are qualitatively identified as being differentially expressed in both tumor types within a time point in the same direction. (XLSX 1528 kb)

Additional file 2: **Figure S1** Responses of CT26 mouse and HCT-116 human colorectal carcinoma cells to radiation. **Figure S2** miR-451a levels in HCT-116 and CT26 cells at different doses of radiation. **Figure S3** miR-451a levels in non-transformed primary cells. **Figure S4** miR-451a levels in HCT-116 in survival fraction studies. **Figure S5** Ectopic expression of miR-451a inhibits proliferation and clonogenic survival of CT26 cells. **Figure S6** Inhibition of miR-451a does not affect proliferation of endothelial cells in response radiation. **Figure S7** Ectopic expression of miR-451a inhibits of HCT-116 cells in combination with radiation and 5-FU.**Figure S8** miR binding site predictions for miR-451a on target mRNAs. **Figure S9** Regulation of miR-451a and target genes in human colorectal cancer. (PPTX 1174 kb)

## Abbreviations

ANOVA: Analysis of Variance
CAB39: Calcium binding protein 39
CRC: Colorectal Cancer
CRT: Chemoradiotherapy
EREG: Epiregulin
HUVECs: Human Umbilical Vein Endothelial Cells
MEX3C: Muscle *ex*cess (Mex)-3 RNA binding Family member C
miRs: MicroRNAs
NHLFs: Normal Human Lung Fibroblasts
RISC: RNA induced silencing complex
TCGA: The Cancer Genome Atlas
