# Supplementary material for: microRNA-451a regulates colorectal cancer proliferation in response to radiation
Source: BMC Cancer. 2018 May 3;18:517. doi: 10.1186/s12885-018-4370-1 (PMC5932766; doi:10.1186/s12885-018-4370-1)
Supplement: Supplementary file 2 — Figure S1 Responses of CT26 mouse and HCT-116 human colorectal carcinoma cells to radiation. Figure S2 miR-451a levels in HCT-116 and CT26 cells at different doses of radiation. Figure S3 miR-451a levels in non-transformed primary cells. Figure S4 miR-451a levels in HCT-116 in survival fraction studies. Figure S5 Ectopic expression of miR-451a inhibits proliferation and clonogenic survival of CT26 cells. Figure S6 Inhibition of miR-451a does not affect proliferation of endothelial cells in response radiation. Figure S7 Ectopic expression of miR-451a inhibits of HCT-116 cells in combination with radiation and 5-FU.Figure S8 miR binding site predictions for miR-451a on target mRNAs. Figure S9 Regulation of miR-451a and target genes in human colorectal cancer. (PPTX 1174 kb) [file 12885_2018_4370_MOESM2_ESM.pptx]

## Slide 1
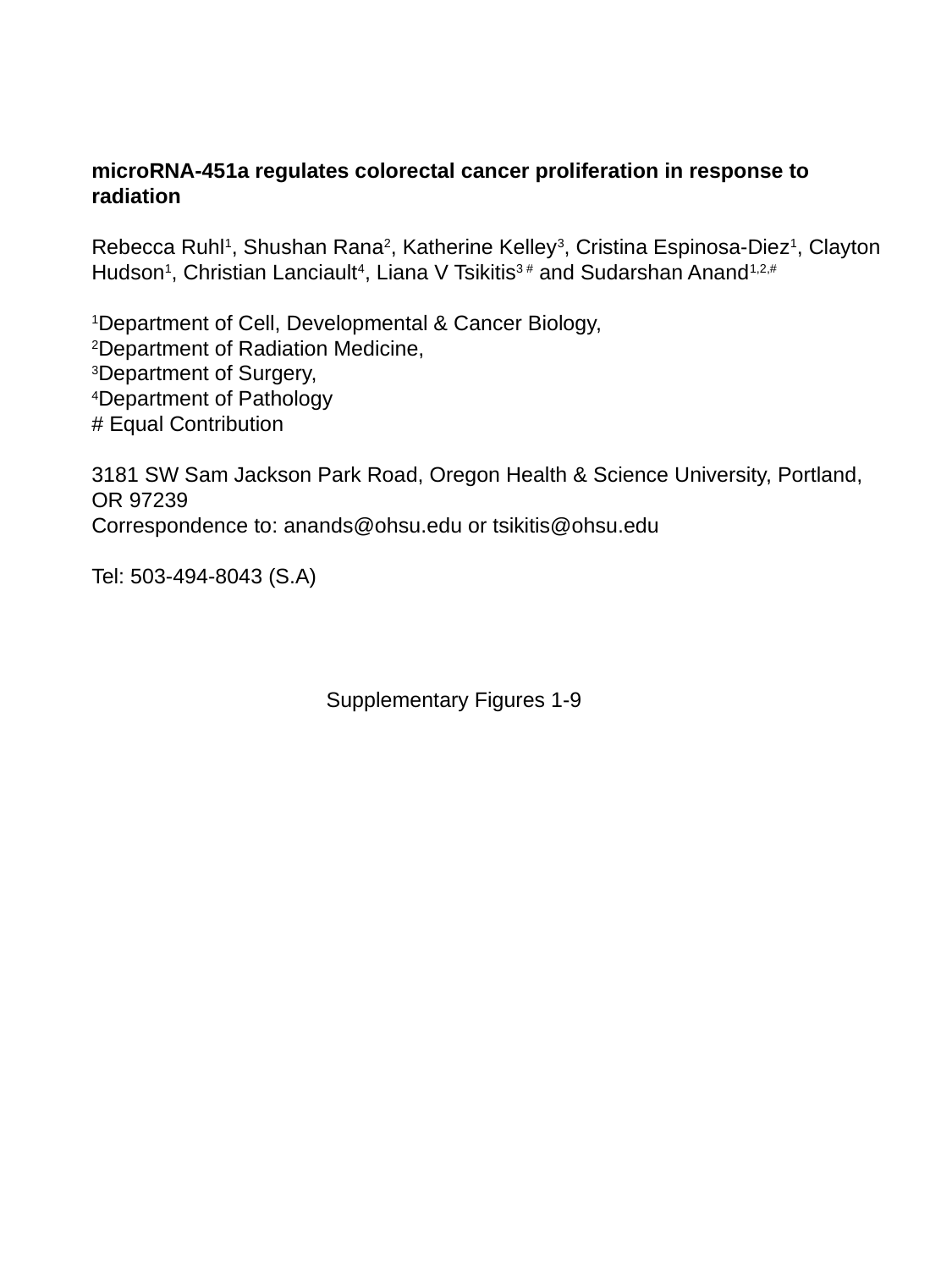

microRNA-451a regulates colorectal cancer proliferation in response to radiation
Rebecca Ruhl1, Shushan Rana2, Katherine Kelley3, Cristina Espinosa-Diez1, Clayton Hudson1, Christian Lanciault4, Liana V Tsikitis3 # and Sudarshan Anand1,2,#
1Department of Cell, Developmental & Cancer Biology,
2Department of Radiation Medicine,
3Department of Surgery,
4Department of Pathology
# Equal Contribution
3181 SW Sam Jackson Park Road, Oregon Health & Science University, Portland, OR 97239
Correspondence to: anands@ohsu.edu or tsikitis@ohsu.edu
Tel: 503-494-8043 (S.A)
Supplementary Figures 1-9

## Slide 2
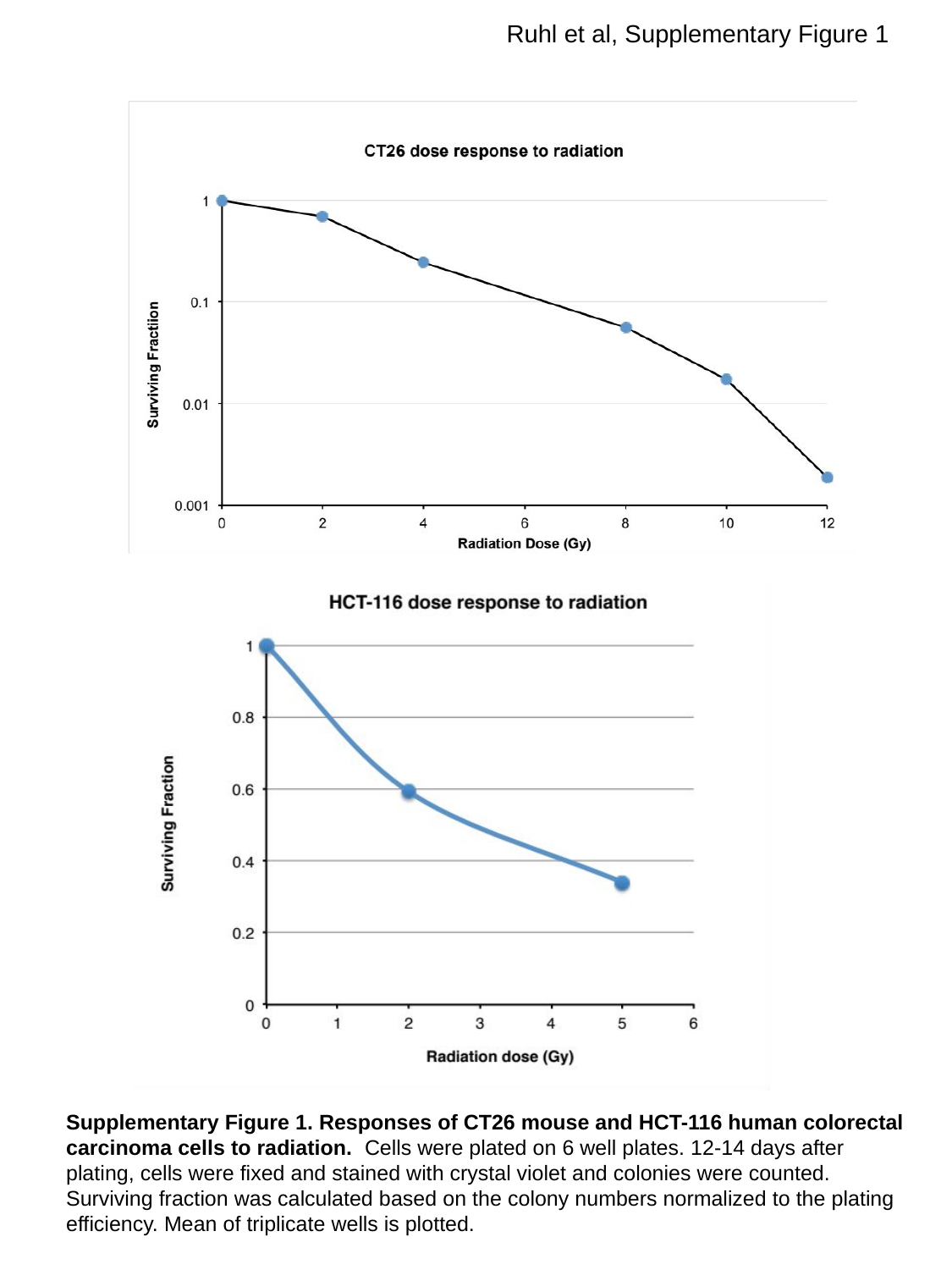

Ruhl et al, Supplementary Figure 1
Supplementary Figure 1. Responses of CT26 mouse and HCT-116 human colorectal carcinoma cells to radiation. Cells were plated on 6 well plates. 12-14 days after plating, cells were fixed and stained with crystal violet and colonies were counted. Surviving fraction was calculated based on the colony numbers normalized to the plating efficiency. Mean of triplicate wells is plotted.

## Slide 3
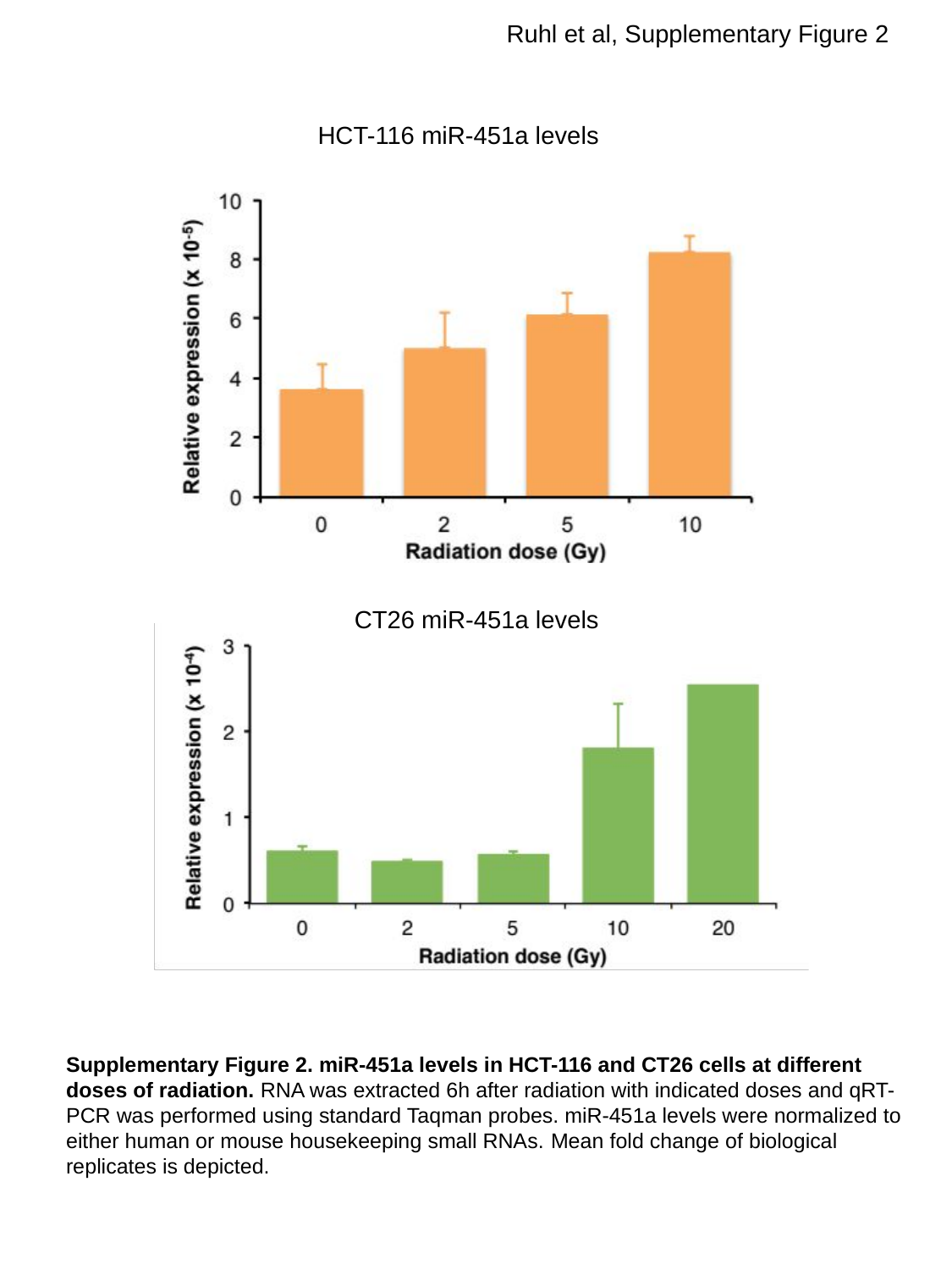

Ruhl et al, Supplementary Figure 2
HCT-116 miR-451a levels
CT26 miR-451a levels
Supplementary Figure 2. miR-451a levels in HCT-116 and CT26 cells at different doses of radiation. RNA was extracted 6h after radiation with indicated doses and qRT-PCR was performed using standard Taqman probes. miR-451a levels were normalized to either human or mouse housekeeping small RNAs. Mean fold change of biological replicates is depicted.

## Slide 4
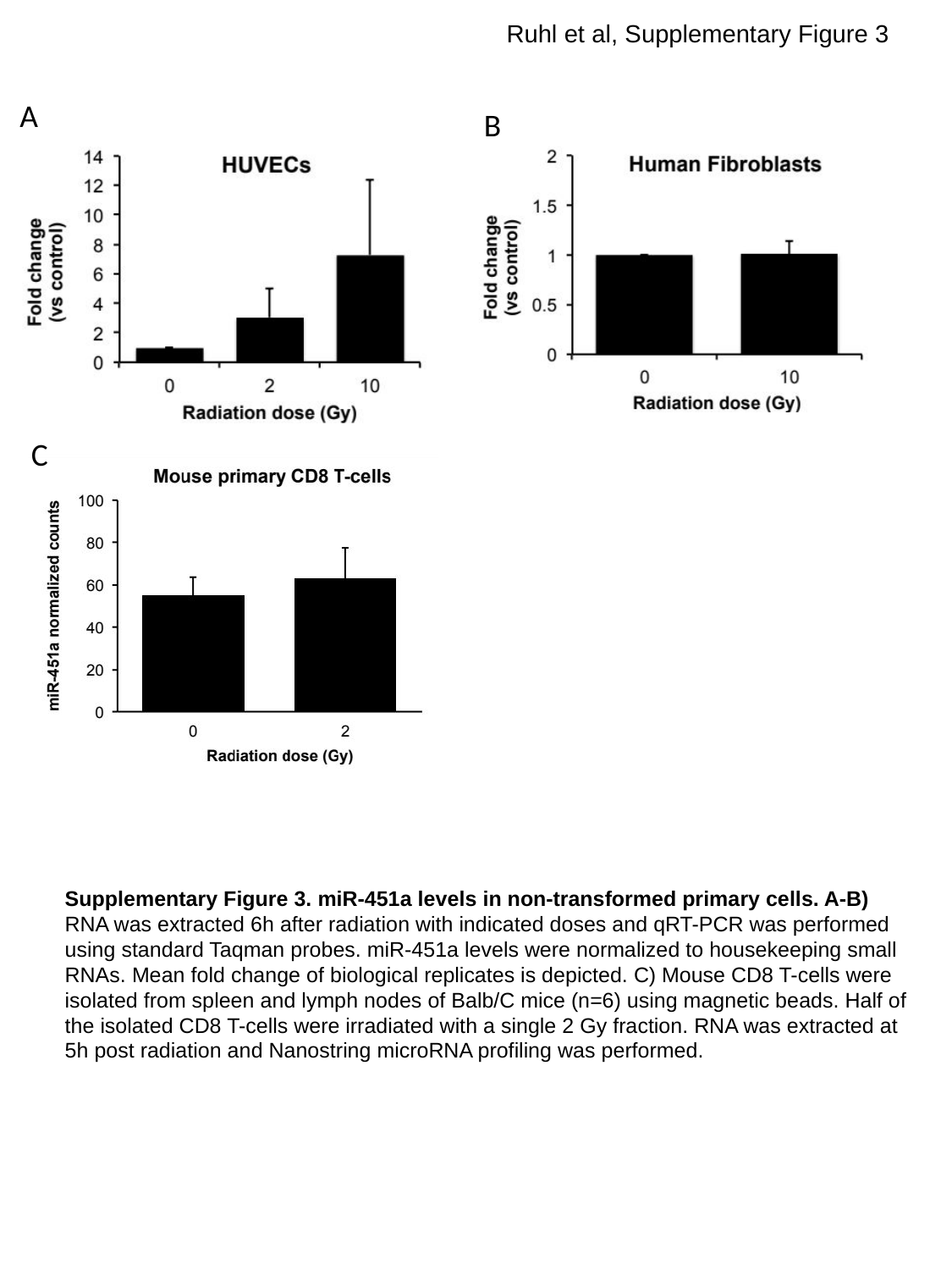

Ruhl et al, Supplementary Figure 3
A
B
C
Supplementary Figure 3. miR-451a levels in non-transformed primary cells. A-B) RNA was extracted 6h after radiation with indicated doses and qRT-PCR was performed using standard Taqman probes. miR-451a levels were normalized to housekeeping small RNAs. Mean fold change of biological replicates is depicted. C) Mouse CD8 T-cells were isolated from spleen and lymph nodes of Balb/C mice (n=6) using magnetic beads. Half of the isolated CD8 T-cells were irradiated with a single 2 Gy fraction. RNA was extracted at 5h post radiation and Nanostring microRNA profiling was performed.

## Slide 5
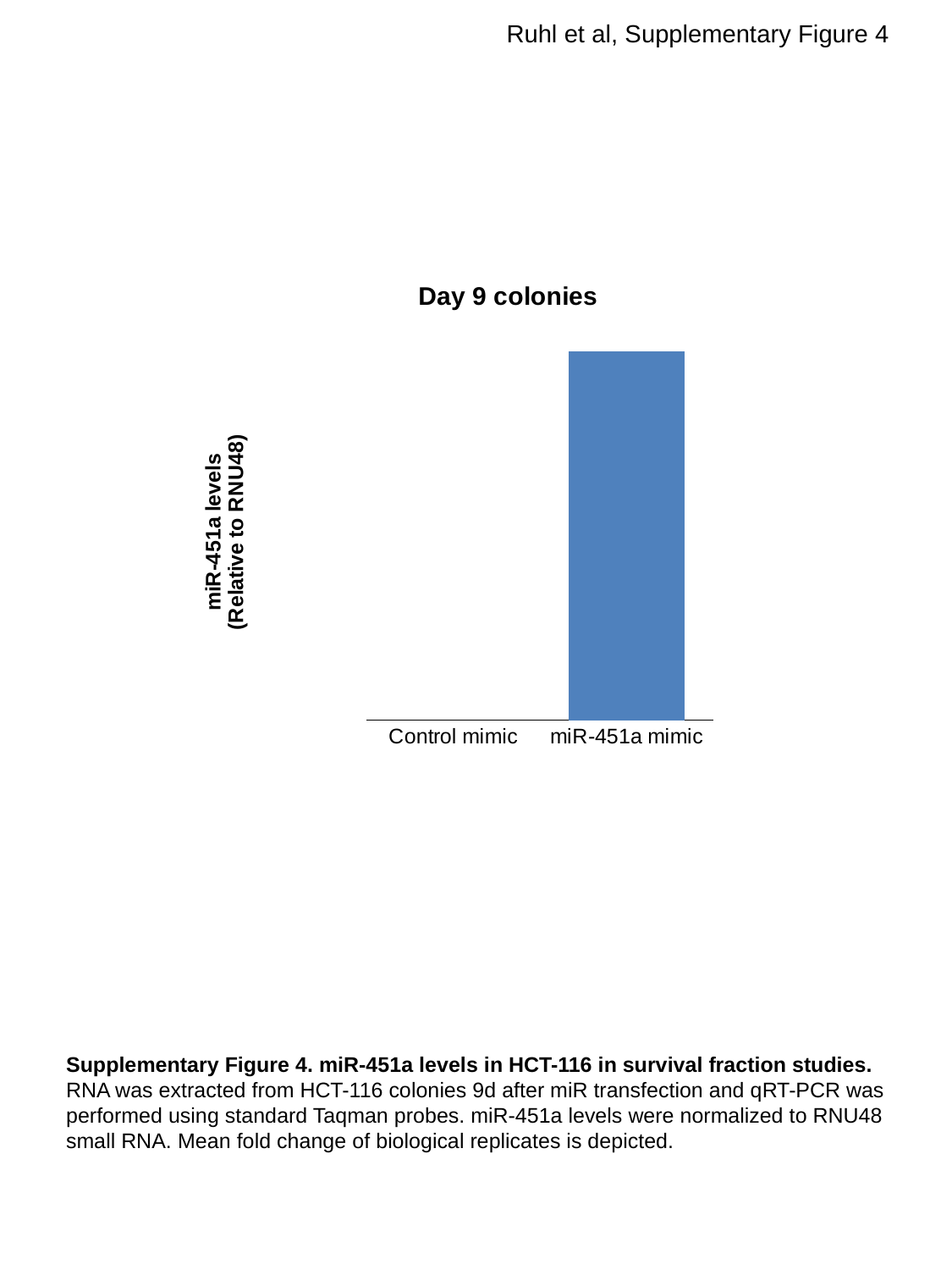

Ruhl et al, Supplementary Figure 4
### Chart: Day 9 colonies
| Category | |
|---|---|
| Control mimic | 1.14691724542343e-05 |
| miR-451a mimic | 0.0292004845212913 |Supplementary Figure 4. miR-451a levels in HCT-116 in survival fraction studies. RNA was extracted from HCT-116 colonies 9d after miR transfection and qRT-PCR was performed using standard Taqman probes. miR-451a levels were normalized to RNU48 small RNA. Mean fold change of biological replicates is depicted.

## Slide 6
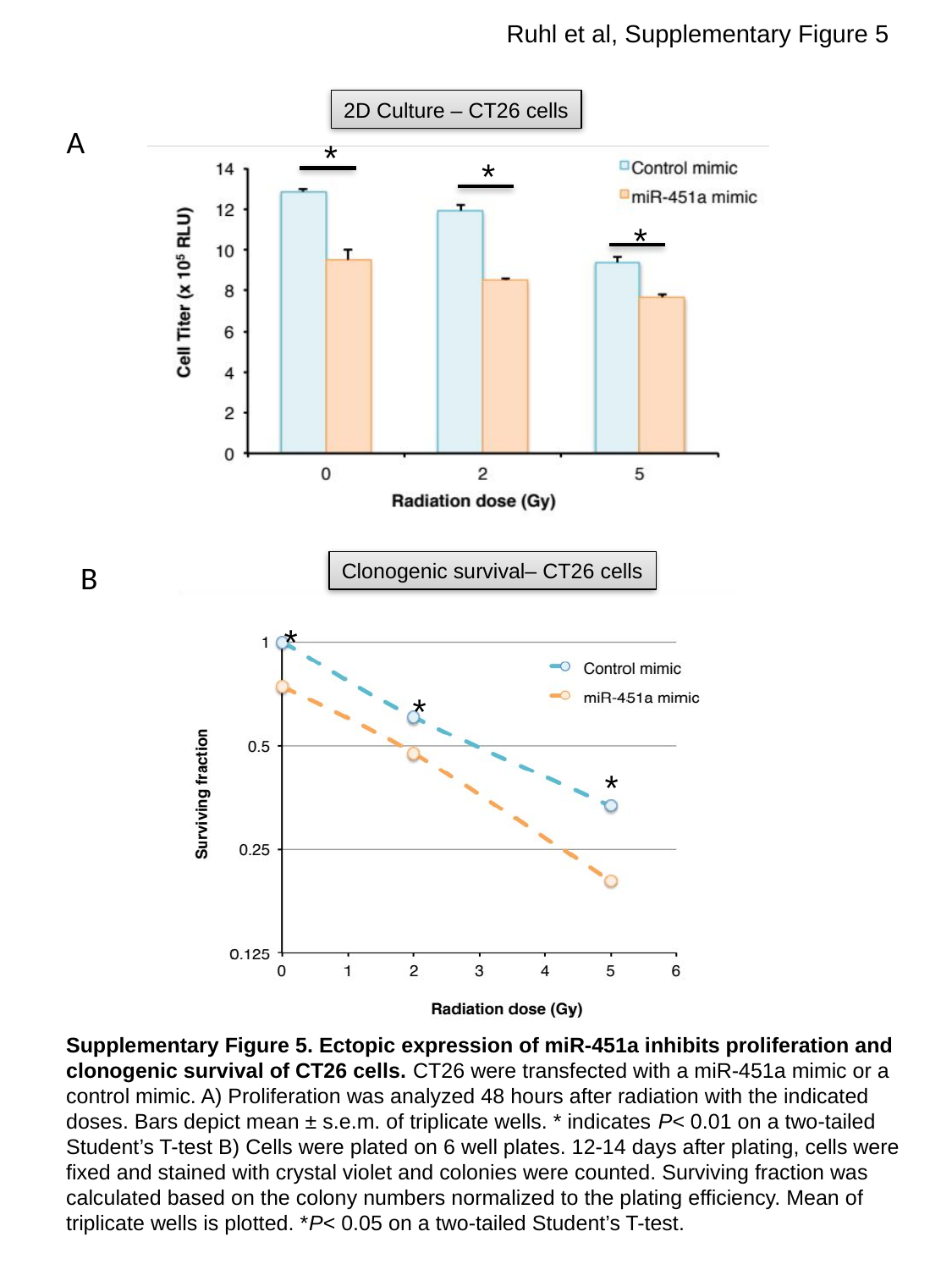

Ruhl et al, Supplementary Figure 5
2D Culture – CT26 cells
A
*
*
*
B
Clonogenic survival– CT26 cells
*
*
*
Supplementary Figure 5. Ectopic expression of miR-451a inhibits proliferation and clonogenic survival of CT26 cells. CT26 were transfected with a miR-451a mimic or a control mimic. A) Proliferation was analyzed 48 hours after radiation with the indicated doses. Bars depict mean ± s.e.m. of triplicate wells. * indicates P< 0.01 on a two-tailed Student’s T-test B) Cells were plated on 6 well plates. 12-14 days after plating, cells were fixed and stained with crystal violet and colonies were counted. Surviving fraction was calculated based on the colony numbers normalized to the plating efficiency. Mean of triplicate wells is plotted. *P< 0.05 on a two-tailed Student’s T-test.

## Slide 7
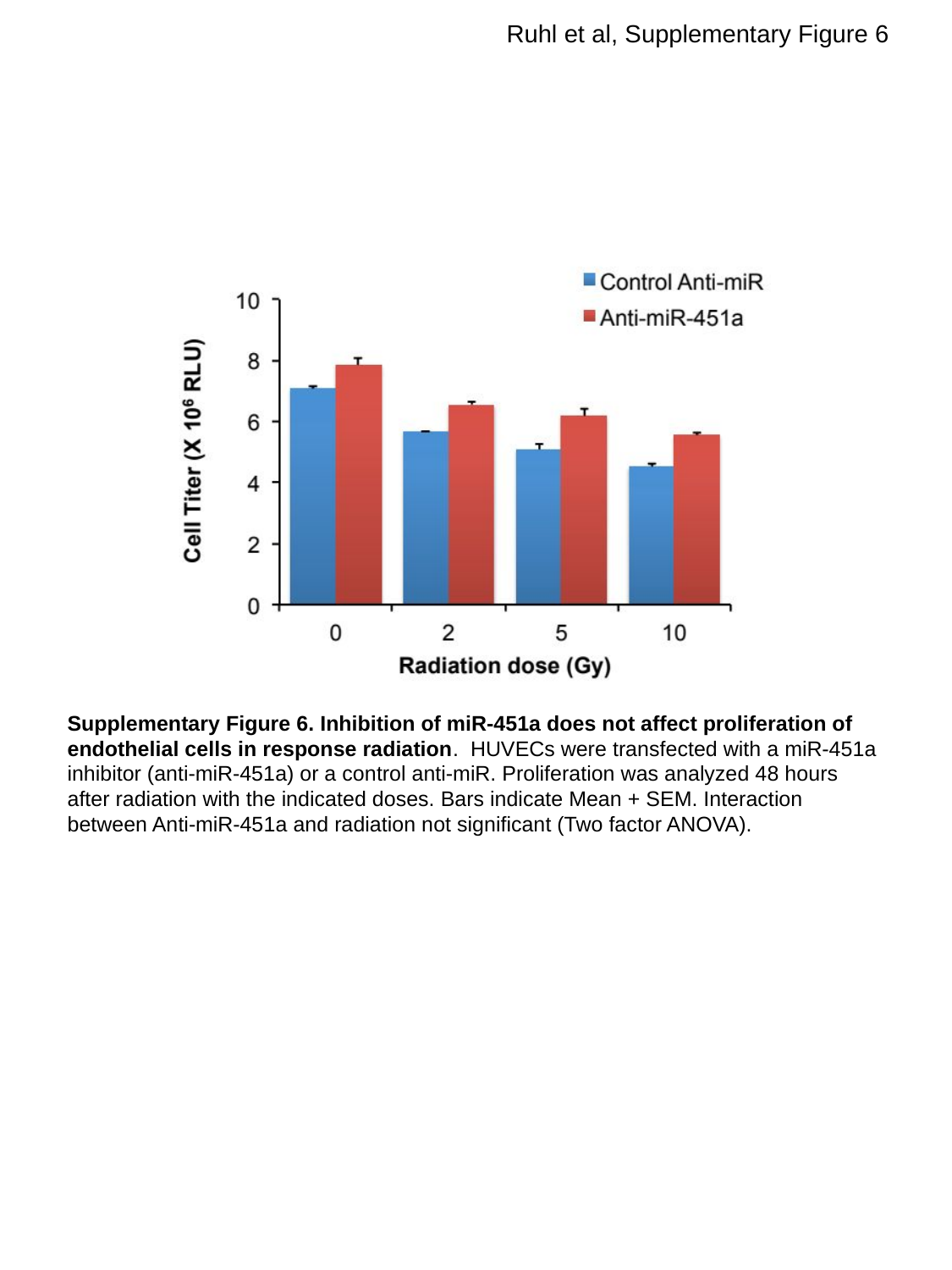

Ruhl et al, Supplementary Figure 6
Supplementary Figure 6. Inhibition of miR-451a does not affect proliferation of endothelial cells in response radiation. HUVECs were transfected with a miR-451a inhibitor (anti-miR-451a) or a control anti-miR. Proliferation was analyzed 48 hours after radiation with the indicated doses. Bars indicate Mean + SEM. Interaction between Anti-miR-451a and radiation not significant (Two factor ANOVA).

## Slide 8
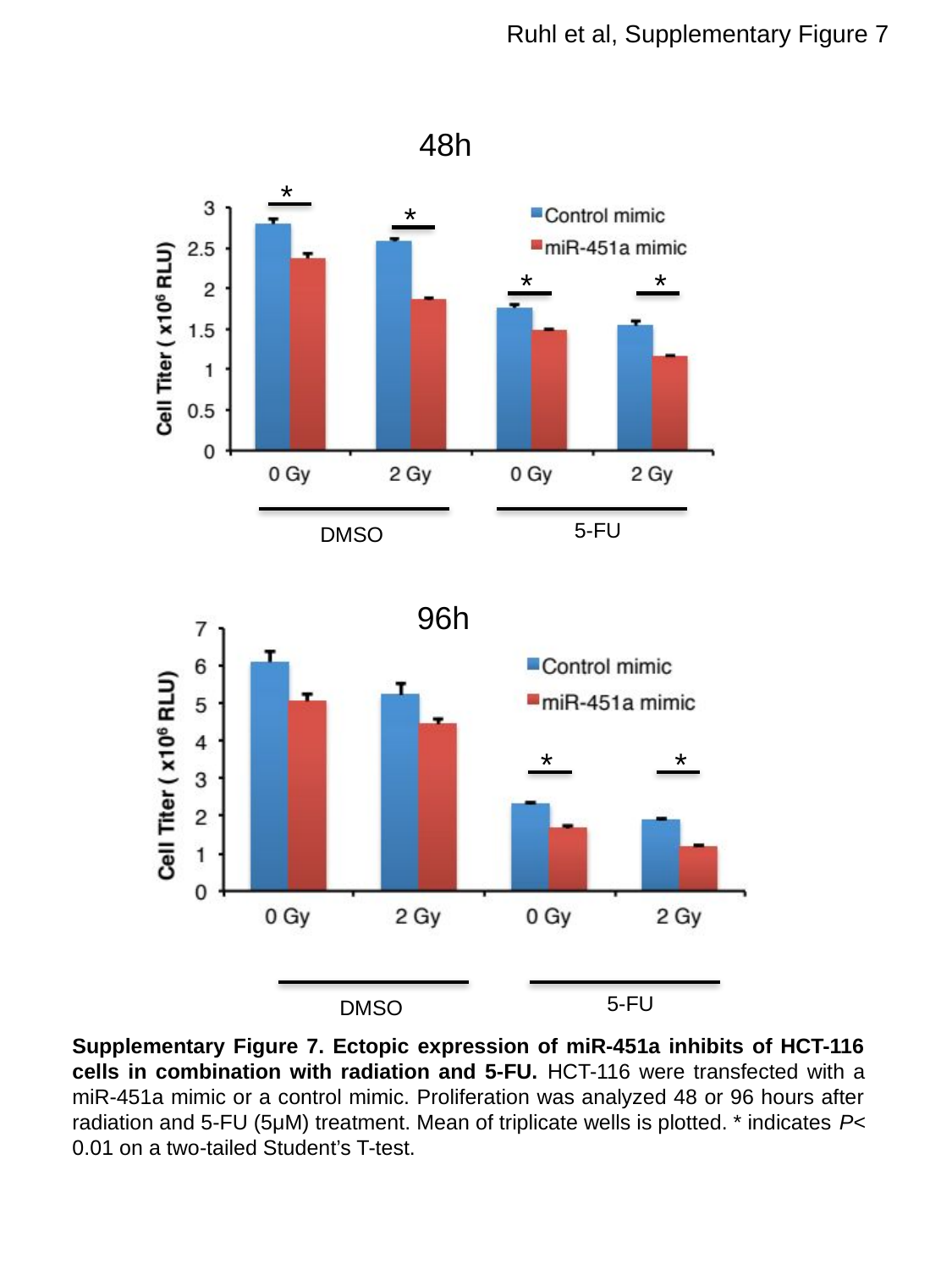

Ruhl et al, Supplementary Figure 7
48h
*
*
*
*
5-FU
DMSO
96h
*
*
5-FU
DMSO
Supplementary Figure 7. Ectopic expression of miR-451a inhibits of HCT-116 cells in combination with radiation and 5-FU. HCT-116 were transfected with a miR-451a mimic or a control mimic. Proliferation was analyzed 48 or 96 hours after radiation and 5-FU (5μM) treatment. Mean of triplicate wells is plotted. * indicates P< 0.01 on a two-tailed Student’s T-test.

## Slide 9
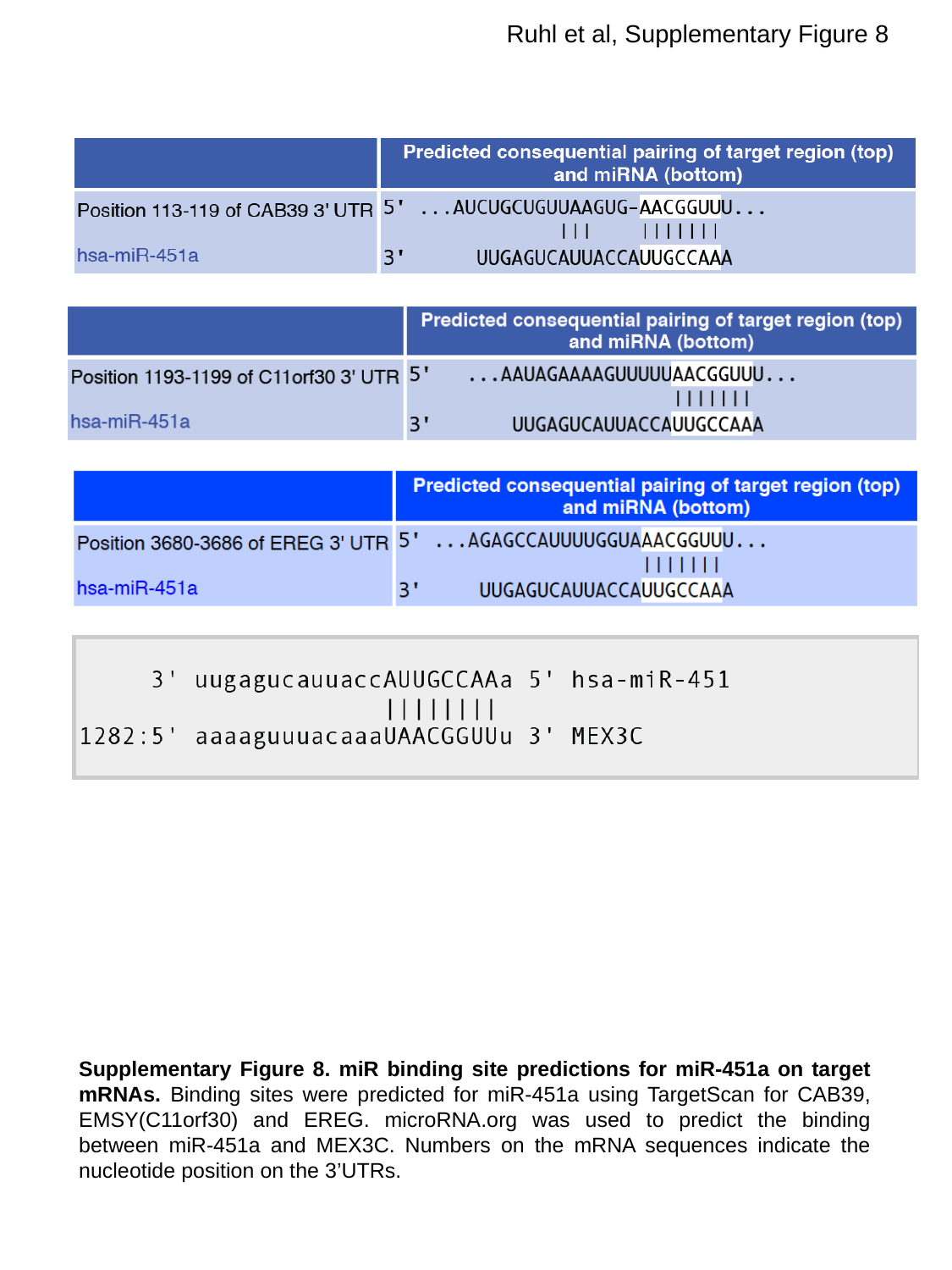

Ruhl et al, Supplementary Figure 8
Supplementary Figure 8. miR binding site predictions for miR-451a on target mRNAs. Binding sites were predicted for miR-451a using TargetScan for CAB39, EMSY(C11orf30) and EREG. microRNA.org was used to predict the binding between miR-451a and MEX3C. Numbers on the mRNA sequences indicate the nucleotide position on the 3’UTRs.

## Slide 10
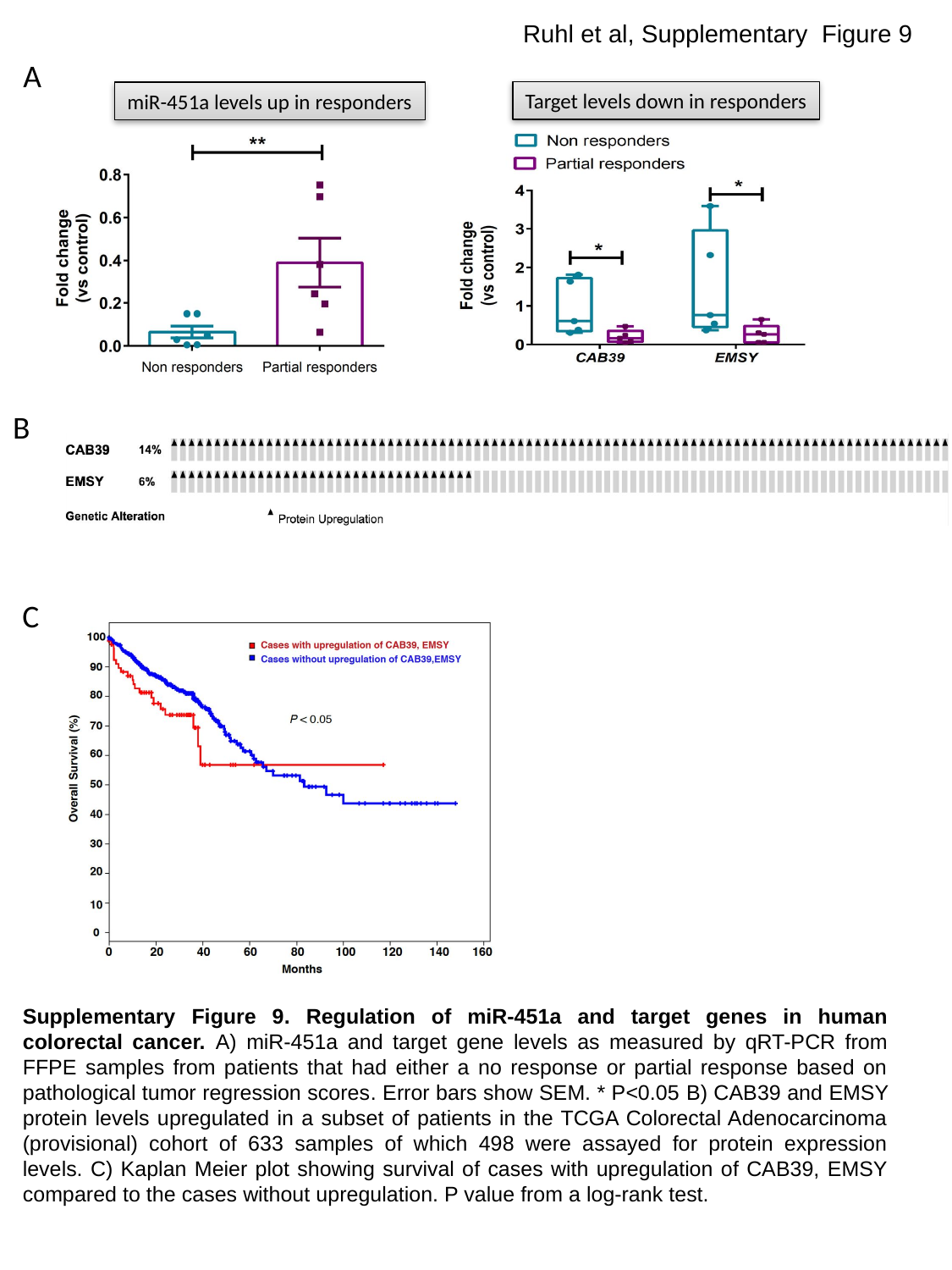

Ruhl et al, Supplementary Figure 9
A
Target levels down in responders
miR-451a levels up in responders
B
C
Supplementary Figure 9. Regulation of miR-451a and target genes in human colorectal cancer. A) miR-451a and target gene levels as measured by qRT-PCR from FFPE samples from patients that had either a no response or partial response based on pathological tumor regression scores. Error bars show SEM. * P<0.05 B) CAB39 and EMSY protein levels upregulated in a subset of patients in the TCGA Colorectal Adenocarcinoma (provisional) cohort of 633 samples of which 498 were assayed for protein expression levels. C) Kaplan Meier plot showing survival of cases with upregulation of CAB39, EMSY compared to the cases without upregulation. P value from a log-rank test.
